# Empirical Antibiotic Initiation for Suspected Early-Onset Neonatal Infection in the Setting of Maternal GBS Screening and Intrapartum Antibiotic Prophylaxis: A Single-Center Retrospective Cohort Study

**DOI:** 10.3390/medicina62101926

**Published:** 2026-10-06

**Authors:** Anna Zmelonek-Znamirowska, Grzegorz Świercz, Jakub Mlodawski, Marta Młodawska, Piotr Kaczmarek

**Affiliations:** 1 Department of Obstetrics and Gynecology, Voivodeship Combined Hospital, 25-736 Kielce, Poland; grzegorz.swiercz@ujk.edu.pl (G.Ś.); jakub.mlodawski@ujk.edu.pl (J.M.); 2 Collegium Medicum, Jan Kochanowski University, 25-369 Kielce, Poland; marta.mlodawska@ujk.edu.pl; 3 Department of Gynecology, Reproductive and Fetal Therapy, Polish Mother’s Memorial Hospital Research Institute, 93-338 Lodz, Poland; kaczmarekpiotr1@gmail.com; 4 Endocrinology and Metabolic Diseases Clinic, Medical University of Lodz, 90-419 Lodz, Poland

**Keywords:** suspected early-onset neonatal infection, culture-confirmed EOS, Group B Streptococcus, intrapartum antibiotic prophylaxis, empirical antibiotic therapy, blood culture, neonatal infection

## Abstract

*Background and Objectives*: Maternal Group B Streptococcus (GBS) screening and intrapartum antibiotic prophylaxis have reduced early-onset GBS disease. However, suspected early-onset neonatal infection (EONI) remains a broader clinical challenge, as signs prompting empirical antibiotic treatment may also occur in non-infectious disorders of postnatal adaptation. Therefore, this study evaluated the frequency of systemic antibiotic initiation for suspected EONI and the utilization and microbiological yield of blood cultures in a single-center cohort managed with routine maternal GBS screening and intrapartum antibiotic prophylaxis. *Materials and Methods*: This retrospective cohort study included 2010 mother–newborn dyads delivered between 2019 and 2022. The primary outcome was systemic antibiotic therapy initiated within the first 72 h of life because of suspected EONI. Blood culture utilization and microbiological yield were analyzed separately from the primary treatment outcome. Positive blood culture findings were interpreted descriptively because of the small number of isolates and the possibility of contamination. *Results*: Empirical systemic antibiotic therapy for suspected EONI was initiated in 71 neonates (3.53%). The observed treatment rates among neonates born to GBS-colonized and non-colonized mothers were 16/402 (3.98%) vs. 55/1608 (3.42%) (RR 1.16, 95% CI 0.67–2.01; *p* = 0.59). In an exploratory multivariable analysis, the adjusted OR for maternal GBS colonization and antibiotic initiation was 1.33 (95% CI 0.68–2.60; *p* = 0.408). Lower gestational age was the strongest associated factor. Treated neonates were more frequently preterm and had more respiratory disorders, NICU admissions, pPROM, and lower Apgar scores. Blood cultures were obtained in 79 neonates, including 63 treated for suspected EONI. Nine sampled neonates had positive blood cultures, including seven treated neonates; several isolates were compatible with contamination. No GBS isolates were detected among the sampled neonates. *Conclusions*: Empirical systemic antibiotics were initiated for suspected EONI in 3.53% of neonates. This treatment frequency does not estimate early-onset sepsis (EOS) incidence. The study did not detect a statistically significant association between maternal GBS colonization and antibiotic initiation, but the wide confidence intervals do not exclude clinically relevant increases in treatment. Selective blood culture sampling, possible contamination, and incomplete microbiological adjudication preclude reliable estimates of culture-confirmed EOS incidence or pathogen distribution.

## 1. Introduction

Intrapartum antibiotic prophylaxis (IAP) has substantially reduced the incidence of early-onset disease caused by Group B Streptococcus (GBS; *Streptococcus agalactiae*) [1,2,3,4,5]. Nevertheless, early-onset sepsis (EOS) remains an important clinical problem, particularly among preterm and clinically unstable neonates. EOS generally refers to neonatal sepsis presenting within the first 48–72 h of life and is commonly related to vertical transmission of microorganisms colonizing the maternal genital tract [6,7,8,9]. In high-income countries, the most frequently discussed pathogens include GBS and *Escherichia coli* [6,10,11].

Epidemiological studies have reported changes in the microbiological pattern of culture-confirmed EOS, including a relatively increasing contribution of Gram-negative organisms, particularly *E. coli* [6,10,11,12]. This observation appears especially relevant among preterm and very low birth weight neonates [6,11,12,13]. It has been suggested that these changes may partly reflect the selective effectiveness of IAP against GBS and the limited activity of some beta-lactam prophylactic regimens against certain Gram-negative organisms [1,3,14,15].

ACOG recommends vaginal–rectal screening for GBS at 36 0/7–37 6/7 weeks of gestation and intrapartum antibiotic prophylaxis when indicated [16]. International recommendations generally identify intravenous penicillin as the first-line agent for prevention of early-onset GBS disease, with ampicillin considered an acceptable alternative [1,14,16,17]. Necessary obstetric interventions should not be delayed solely to achieve at least 4 h of prophylaxis before delivery [16]. However, clinical practice may differ across institutions because of local antimicrobial policies, drug availability, allergy management, and regional resistance patterns [1,14,16,17]. Variation in screening-based and risk-based strategies limits direct comparison of data between countries and centers [3,18,19].

In routine neonatal practice, antibiotic therapy is frequently initiated empirically because early symptoms of infection are nonspecific and may overlap with non-infectious postnatal adaptation disorders, especially in preterm infants or neonates with respiratory compromise. Perinatal risk factors such as pPROM, low Apgar scores, and clinical instability requiring NICU admission may also influence the threshold for empirical treatment [6,7,20,21]. In neonatal care, empirical antibiotic initiation represents an important process-of-care measure because neonatal antibiotic exposure has implications for antimicrobial stewardship and may influence early-life microbiome development [6,22,23].

Neonatal assessment combines maternal and intrapartum risk factors with the infant’s evolving clinical condition. For infants born at ≥35 weeks, AAP guidance permits categorical risk assessment, multivariable risk estimation, or serial physical examinations; preterm infants require a gestation-specific approach [24,25]. NICE NG195, updated in May 2026, recommends prompt empirical treatment for any red-flag indicator or at least two other risk factors or clinical indicators, while allowing clinical judgment and observation for an isolated non-red-flag finding [26].

Blood culture should be obtained before the first neonatal antibiotic dose [26]. First-line empirical regimens differ across guidelines: AAP recommends ampicillin plus gentamicin for most infants, whereas NICE recommends benzylpenicillin plus gentamicin unless local resistance requires an alternative [24,25,26]. Polish Neonatal Society standards also recommend ampicillin plus gentamicin for empirical treatment, with the doses and dosing intervals adjusted to gestational age and birth weight [27]. AAP recommends discontinuation at 36–48 h of sterile culture incubation unless there is evidence of site-specific infection [24,25]. NICE recommends stopping at 36 h when cultures are negative, initial suspicion is not strong, the infant is clinically reassuring, and CRP levels and trends are reassuring [26].

Blood culture remains the reference method for identifying invasive infection, but its diagnostic yield may be influenced by blood sample volume, prior antibiotic exposure, and sampling practices [9,11,12]. Conversely, microbial growth may represent contamination, particularly when repeat cultures, time to positivity, and complete case-level microbiological adjudication are unavailable. These limitations should also be considered when interpreting pathogen distribution [2,11,12,28]. The 2024 Swiss guideline emphasizes adequate pre-antibiotic sampling, including at least 1 mL of blood, and early reassessment to limit unnecessary exposure [29].

Despite extensive multicenter and population-based literature on EOS and early-onset GBS disease, detailed data describing local empirical treatment practices and blood culture utilization remain limited in some Central and Eastern European settings [10,11,12,28,30].

Accordingly, the primary objective of this study was to compare the frequency of systemic antibiotic initiation within the first 72 h of life for suspected early-onset neonatal infection (EONI) between neonates born to GBS-colonized and non-colonized mothers. Secondary objectives were to describe blood culture utilization and microbiological yield during evaluation for suspected EONI and to identify maternal and perinatal factors associated with antibiotic initiation. Among GBS-positive mothers, treatment frequency was additionally described according to the timing-defined completeness of IAP.

## 2. Materials and Methods

### 2.1. Study Design and Population

This retrospective cohort study was conducted at the Department of Obstetrics and Gynecology of the Voivodeship Combined Hospital in Kielce, Poland. Initially, 2099 mother–newborn dyads delivered between June 2019 and August 2022 were assessed for eligibility. The study cohort consisted of cases with available prenatal screening documentation and a documented maternal microbiological screening result for *Streptococcus agalactiae* colonization.

**Figure 1:**
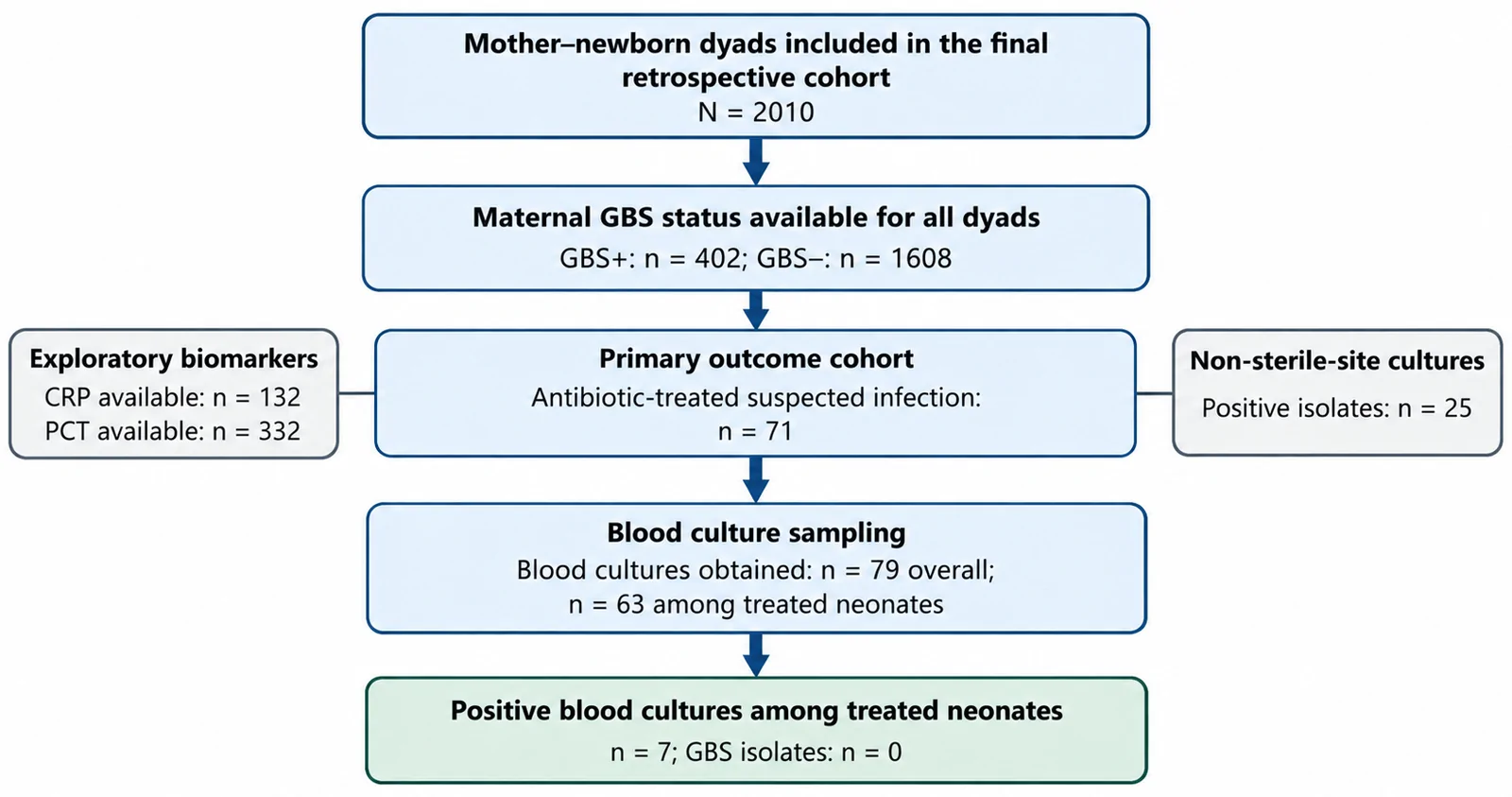
Study flowchart and analytic cohorts. Note: biomarker and non-sterile-site culture analyses were exploratory and restricted to neonates with available data. The zero GBS-isolate count in the flowchart refers to blood cultures obtained from sampled treated neonates, not to the absence of GBS EOS in the overall cohort. Counts by individual exclusion criteria and their possible overlap were unavailable; the listed criteria should not be interpreted as mutually exclusive or sequential exclusion counts.

Exclusion criteria were the absence of a documented maternal GBS culture result, extreme prematurity (birth before 28 weeks of gestation), multiple pregnancy, abnormal prenatal or postnatal genetic test results, and neonatal anatomical abnormalities potentially causing respiratory disorders. A total of 89 mother–newborn dyads were excluded, leaving 2010 in the final analytic cohort. Counts for individual exclusion criteria were unavailable, so exclusions are presented in aggregate rather than as a quantified sequence of screening steps. A study flowchart illustrating patient selection and cohort derivation is presented in Figure 1.
Assessed for eligibility: 2099 mother–newborn dyadsExcluded: 89 dyads in total • Unknown maternal GBS culture result • Extreme prematurity (<28 weeks) • Multiple pregnancy • Abnormal prenatal or postnatal genetic test results • Neonatal anatomical abnormalities potentially causing respiratory disorders Counts by individual criteria unavailableIncluded: 2010 mother–newborn dyads  Analytic cohorts shown below

### 2.2. Intrapartum Antibiotic Prophylaxis

IAP was administered according to the local institutional protocol. During the study period, intravenous cefuroxime was used as the standard prophylactic agent at the study center. The institutional regimen specified an initial intravenous dose of 2 g cefuroxime after transfer to the delivery room and a repeat intravenous dose of 1 g after 6 h of ongoing labor. Complete IAP was defined as prophylaxis initiated at least 4 h before delivery, whereas incomplete IAP was defined as prophylaxis initiated less than 4 h before delivery. These categories describe the interval from initiation of prophylaxis to delivery, not verified adherence to the full dosing regimen or guideline-defined adequacy of cefuroxime prophylaxis.

Although the institutional dosing regimen was known, individual-level documentation of the doses actually administered, repeat intrapartum dosing, allergy-related modifications of prophylaxis, and adherence to the institutional IAP protocol was not consistently available in the retrospective medical records.

### 2.3. Data Collection

Clinical data were obtained from the Astraia electronic system, the hospital electronic medical record system (Asseco Medical Management Solutions, AMMS), and paper-based medical records, including maternal and neonatal charts, delivery logs, and documentation from the neonatal ward.

Collected maternal and obstetric variables included maternal age, maternal body weight, selected comorbidities, GBS screening result, IAP implementation, premature rupture of membranes (PROM), preterm premature rupture of membranes (pPROM), duration from membrane rupture to delivery, suspected intrauterine infection, gestational age at delivery, and mode of delivery. Neonatal data included birth weight, Apgar scores, umbilical cord blood gas analysis, respiratory disorders, NICU admission, length of hospitalization, inflammatory biomarkers, microbiological culture results, neonatologist-diagnosed neonatal infection, and antibiotic therapy.

### 2.4. Microbiological Diagnostics

Screening for *Streptococcus agalactiae* colonization was routinely performed between 35 and 37 weeks of gestation in accordance with national guidelines. In women admitted with preterm labor or threatened preterm labor before 35 weeks, screening was performed upon admission. Specimens were collected from the lower vagina and rectum and transported to the microbiology laboratory in appropriate transport media. Isolation of *S. agalactiae* was performed using standard culture-based methods, and antimicrobial susceptibility testing was performed when clinically applicable.

Evaluation for suspected EONI and the decision to initiate empirical treatment were made by the attending neonatologist on the basis of the infant’s clinical condition and relevant perinatal risk factors, in accordance with the standards of neonatal medical care in Poland [27]. Blood cultures were obtained when EONI was suspected, according to clinical indications and the local neonatal protocol. Culture testing was performed using conventional microbiological techniques. The empirical neonatal antibiotic regimen consisted of ampicillin plus gentamicin; doses and dosing intervals were adjusted to gestational age and birth weight in accordance with the Polish Neonatal Society recommendations [27]. Detailed microbiological parameters, including blood culture volume, time to positivity, repeat culture results, anaerobic culture availability, and complete case-level microbiological adjudication, were not consistently available in the retrospective dataset. Additional cultures obtained from non-sterile sites were analyzed separately and were not used to define invasive infection or EOS etiology.

### 2.5. Study Outcomes and Definitions

The primary outcome was empirical systemic antibiotic initiation within the first 72 h of life because of suspected EONI. The indication for antibiotic initiation was ascertained retrospectively from physicians’ clinical notes in AMMS, which were reviewed to establish whether treatment was initiated because of suspected infection. The indication was not inferred solely from antibiotic administration or recorded diagnoses. Although clinical assessment incorporated the infant’s condition and relevant perinatal risk factors, the retrospective records did not permit reliable reconstruction of a uniform item-by-item decision algorithm for every neonate. This operational definition captured the clinical decision to initiate treatment, irrespective of subsequent blood culture findings, and did not require microbiological confirmation. Throughout this manuscript, “suspected EONI” denotes the clinical suspicion prompting evaluation or empirical treatment within this 72-h window; it does not denote established sepsis. Shortened treatment labels in the tables and flowchart refer to the same primary outcome.

Secondary outcomes included neonatologist-diagnosed neonatal infection, blood culture sampling within the first 72 h of life, positive blood culture during evaluation for suspected EONI, and antibiotic initiation for suspected EONI without a positive blood culture. “Neonatologist-diagnosed neonatal infection” denotes a diagnosis entered in the AMMS electronic medical record system. This variable was ascertained separately from antibiotic initiation and was not treated as culture-confirmed EOS. The category without a positive blood culture included both sampled neonates without growth and neonates without blood culture sampling; it therefore did not uniformly represent a negative culture result.

A positive blood culture denoted reported microbial growth. “Culture-confirmed EOS” denotes EOS supported by a clinically significant blood culture isolate, rather than growth attributable to contamination. Isolates typically associated with skin or environmental contamination were classified as presumed contaminants or isolates compatible with contamination.

Exploratory analyses of inflammatory biomarkers and isolates obtained from non-sterile neonatal sites were not included in the primary or secondary study objectives and were analyzed separately as descriptive exploratory findings. Biomarker comparisons were hypothesis-generating available-case analyses of selectively tested neonates, not a systematic assessment of neonatal inflammation. CRP > 10 mg/L and PCT > 2 ng/mL were used as fixed descriptive thresholds. The PCT analysis did not account for exact postnatal age at sampling or use age-specific thresholds. A subgroup without systemic antibiotic therapy and without a recorded neonatologist diagnosis of infection was examined descriptively; these selection criteria do not ensure the absence of infection or other inflammatory stimuli.

### 2.6. Statistical Analysis

Continuous variables were summarized as mean ± standard deviation (SD) or median with interquartile range (IQR), depending on distribution. Categorical variables were presented as absolute counts and percentages. The distribution of continuous variables was assessed using visual inspection of histograms and Q-Q plots and, where appropriate, the Shapiro–Wilk test.

Between-group comparisons were performed using the chi-square test or Fisher’s exact test for categorical variables, depending on expected cell counts. Continuous variables were compared using the Student’s *t*-test for approximately normally distributed variables and the Mann–Whitney U test for non-normally distributed variables. For comparisons involving more than two groups, the Kruskal–Wallis test was used, followed by Dunn’s post hoc test where applicable.

Effect estimates were reported as risk ratios (RRs) with 95% confidence intervals (CIs) for unadjusted group comparisons. Agreement between antibiotic initiation for suspected EONI and neonatologist-diagnosed neonatal infection was assessed descriptively using cross-tabulation. Interpretation of the unadjusted RRs and adjusted odds ratios (ORs) emphasizes the point estimates and their 95% CIs; a non-significant *p*-value does not establish the absence of an association or equivalence between groups.

An exploratory multivariable logistic regression model was constructed to assess whether maternal GBS colonization was independently associated with systemic antibiotic initiation for suspected EONI after adjustment for clinically relevant covariates available in the retrospective dataset. Candidate variables were selected a priori based on their clinical relevance to antibiotic initiation and data availability, rather than on statistical significance in univariable analyses. The final model included maternal GBS colonization status, gestational age at delivery, pPROM, suspected intrauterine infection, hypertensive disorders of pregnancy, and cesarean delivery. The number of covariates was restricted to reduce the risk of overfitting given the limited number of outcome events. The model was intended to estimate adjusted associations with antibiotic initiation and not to develop a prediction model or identify causal determinants of culture-confirmed EOS.

Neonatal respiratory disorders, NICU admission, and length of hospitalization were not included as independent predictors in the multivariable model because they may have been part of the clinical presentation leading to antibiotic initiation or markers of subsequent clinical course rather than baseline confounders.

Positive blood culture outcomes were analyzed descriptively only because blood culture-positive cases were rare and several isolates were compatible with contamination. No multivariable model was constructed for positive blood culture outcomes.

Exploratory analyses were restricted to neonates with available measurements. Missing data were not imputed, and denominators were reported separately for each analysis.

A two-sided *p*-value <0.05 was considered statistically significant. Because the study was exploratory and several secondary analyses were descriptive, *p*-values were not adjusted for multiple comparisons and should be interpreted cautiously. Statistical analyses were performed using R software, version 4.3.1.

## 3. Results

### 3.1. Study Population

A total of 2010 mother–newborn dyads were included in the final analysis. Maternal GBS colonization was documented in 402 women (20.0%), whereas 1608 women (80.0%) were GBS-negative.

Baseline maternal, obstetric, and neonatal characteristics according to maternal GBS colonization status are presented in Table 1.

Continuous variables are presented as mean ± SD or median (IQR), depending on distribution. Categorical variables are presented as *n/N* (%). Percentages were calculated using available data.

### 3.2. Empirical Systemic Antibiotic Initiation for Suspected EONI

Systemic antibiotic therapy within the first 72 h of life because of suspected EONI was initiated in 71 neonates, corresponding to 3.53% of the cohort.

Neonatologist-diagnosed neonatal infection was recorded in 85 of 2009 neonates with available data (4.23%). Among the 71 neonates treated with systemic antibiotics, 70 also had neonatologist-diagnosed neonatal infection. One neonate received antibiotic therapy despite the absence of recorded neonatologist-diagnosed neonatal infection. Conversely, 15 neonates had neonatologist-diagnosed neonatal infection recorded but did not receive systemic antibiotic therapy within the first 72 h of life.

### 3.3. Primary Outcome According to Maternal GBS Colonization Status

Table 2 presents treatment frequencies according to maternal GBS colonization status.

The RR for systemic antibiotic initiation for suspected EONI in the GBS-positive versus GBS-negative group was 1.16 (95% CI 0.67–2.01; *p* = 0.59). The interval included both a lower treatment risk and a clinically relevant increase of up to approximately twofold; thus, the study did not detect a statistically significant association but did not establish the absence of an association. For the additional recorded clinical outcome, neonatologist-diagnosed neonatal infection was recorded in 20/402 neonates born to GBS-positive mothers and in 65/1607 neonates born to GBS-negative mothers with available data. The corresponding frequencies were 4.98% and 4.04%, respectively (RR 1.23, 95% CI 0.75–2.01; *p* = 0.41).

The primary outcome was systemic antibiotic therapy initiated within the first 72 h of life because of suspected early-onset neonatal infection (EONI). This outcome should not be interpreted as culture-confirmed EOS.

### 3.4. Intrapartum Antibiotic Prophylaxis Among GBS-Positive Mothers

Table 3 presents timing-defined IAP categories and neonatal antibiotic initiation among GBS-positive mothers. Because the no-IAP subgroup was very small, these comparisons were descriptive and cannot establish prophylactic effectiveness.

### 3.5. Clinical Characteristics According to Empirical Antibiotic Initiation

Maternal, obstetric, and neonatal characteristics according to empirical antibiotic initiation for suspected EONI are presented in Table 4. Compared with untreated neonates, treated neonates were born at a lower gestational age, were more frequently preterm, and had lower birth weight and lower Apgar scores. The treated group also had more frequent pPROM, hypertensive disorders of pregnancy, umbilical cord pH < 7.25, respiratory disorders, and NICU admission, as well as longer hospitalization. These are characteristics associated with the treated group, not evidence that infection caused these differences.

### 3.6. Blood Culture Sampling and Positive Blood Cultures

Blood cultures were obtained in 79 neonates, corresponding to 3.93% of the total cohort. Among the 71 neonates treated with systemic antibiotics for suspected EONI, blood cultures were obtained in 63 cases. Eight treated neonates did not have a blood culture obtained; they are not classified as culture-negative.

Positive blood cultures were identified in 9 of the 79 neonates who had blood cultures obtained: 7/63 neonates who received systemic antibiotics for suspected EONI and 2/16 neonates who did not receive such treatment. No GBS isolates were detected among the sampled neonates. This observation does not establish that GBS EOS was absent in the overall cohort.

Several isolates were compatible with contamination. The microorganisms identified in the nine neonates with positive blood cultures and their clinical interpretation are presented in Table 5. 

**Table 5:** Microorganisms reported in the nine neonates with positive blood cultures.

Pathogen/Isolate	*n*	Interpretation
*Staphylococcus epidermidis*	3	Isolate compatible with contamination
*Staphylococcus haemolyticus*	1	Isolate compatible with contamination
*Staphylococcus hominis*	1	Isolate compatible with contamination
*Bacillus* sp.	1	Isolate compatible with contamination
*Streptococcus sanguinis*/Gram-positive cocci	1	Possible pathogen
*Granulicatella elegans*/*Corynebacterium* species	1	Possible pathogen or contaminant
*Micrococcus luteus*	1	Isolate compatible with contamination

The table presents blood culture results in nine neonates rather than the number of individual microorganisms. Seven of these neonates received systemic antibiotics for suspected early-onset neonatal infection, whereas two did not. The two positive cultures among untreated neonates yielded *Staphylococcus epidermidis* and *Micrococcus luteus*. The laboratory report “*Granulicatella elegans*/*Corynebacterium* species” refers to one positive culture result in one neonate.

### 3.7. Exploratory Analyses

Microbiological cultures from non-sterile neonatal sites were obtained in selected neonates according to clinical indications. Positive cultures from non-sterile sites were identified in 25 neonates. Of these, 23 neonates received systemic antibiotic therapy within the first 72 h of life, whereas 2 neonates did not. The most frequent specimen source was the rectal swab. Positive cultures were also obtained from conjunctival swabs and endotracheal tube samples. Microorganisms isolated from non-sterile sites included *Enterococcus faecalis*, *Escherichia coli*, *Klebsiella oxytoca*, *Klebsiella pneumoniae*, coagulase-negative staphylococci, *Pseudomonas aeruginosa*, and *Enterobacter cloacae complex*.

CRP values were available in 132/2010 neonates (6.57%). Elevated CRP, defined as >10 mg/L, was observed in 78/132 neonates (59.1%) with available measurements. PCT values were available in 332/2010 neonates (16.52%). Elevated PCT, defined as >2 ng/mL, was observed in 211/332 neonates (63.6%) with available measurements.

An additional descriptive comparison by maternal GBS status was restricted to tested neonates without systemic antibiotic therapy and without neonatologist-diagnosed neonatal infection. In this subgroup, CRP values were available in 99 neonates. Elevated CRP was observed in 56/99 neonates (56.6%). The frequency of elevated CRP did not differ significantly according to maternal GBS colonization status: 29/50 neonates (58.0%) in the GBS-positive group and 27/49 neonates (55.1%) in the GBS-negative group (*p* = 0.77).

In the same subgroup, PCT values were available in 265 neonates. Elevated PCT was observed in 148/265 neonates (55.8%). Elevated PCT was recorded in 73/155 neonates (47.1%) born to GBS-positive mothers and in 75/110 neonates (68.2%) born to GBS-negative mothers (*p* < 0.001). This nominal *p*-value was not adjusted for multiple comparisons.

These selectively obtained findings are hypothesis-generating and establish neither invasive infection nor a relationship between maternal GBS colonization and neonatal inflammation.

### 3.8. Multivariable Analysis

After adjustment for gestational age at delivery, pPROM, suspected intrauterine infection, hypertensive disorders of pregnancy, and cesarean delivery, the adjusted OR for maternal GBS colonization and systemic antibiotic initiation for suspected EONI was 1.33 (95% CI 0.68–2.60; *p* = 0.408). The results of the multivariable logistic regression analysis are presented in Table 6. 

**Table 6:** Exploratory multivariable logistic regression of factors associated with antibiotic initiation for suspected EONI.

Variable	Adjusted OR	95% CI	*p*-Value
Maternal GBS colonization	1.33	0.68–2.60	0.408
Gestational age at delivery, per additional week	0.41	0.35–0.49	<0.001
Preterm premature rupture of membranes	0.93	0.39–2.24	0.879
Suspected intrauterine infection	2.53	0.89–7.16	0.080
Hypertensive disorders of pregnancy	0.60	0.24–1.51	0.282
Cesarean delivery	1.09	0.60–1.99	0.772

Gestational age at delivery showed the strongest association with antibiotic initiation. Each additional week of gestation was associated with lower odds of receiving systemic antibiotics for suspected EONI (adjusted OR 0.41, 95% CI 0.35–0.49; *p* < 0.001). Suspected intrauterine infection showed a non-significant trend toward higher odds of antibiotic initiation for suspected EONI (adjusted OR 2.53, 95% CI 0.89–7.16; *p* = 0.080).

Model summary: complete-case analysis, *n* = 2008; outcome events = 71. Two dyads were excluded because of missing covariate data: one had incomplete information for a component of hypertensive disorder classification, and one had missing information on cesarean delivery status. The outcome was initiation of systemic antibiotic therapy within the first 72 h of life because of suspected early-onset neonatal infection (EONI).

## 4. Discussion

The principal finding was that empirical systemic antibiotics for suspected EONI were initiated in 71 of 2010 neonates (3.53%). This proportion measures local treatment practice, not EOS incidence. The estimates for maternal GBS colonization and antibiotic initiation were RR 1.16 (95% CI 0.67–2.01) and adjusted OR 1.33 (95% CI 0.68–2.60). Both point estimates were above 1, but the intervals included 1 and were too wide to distinguish no association from clinically relevant increases in treatment. Lower gestational age was the strongest associated factor.

The RR describes relative treatment risk, whereas the adjusted OR describes relative odds and should not be read as an adjusted risk ratio. Separately, the treatment endpoint and management within a screened population receiving IAP prevent these findings from establishing that maternal GBS colonization is unrelated to EOS.

The strong association between lower gestational age and antibiotic initiation suggests that the primary outcome reflected treatment decisions in a heterogeneous group whose clinical presentation may have been influenced by prematurity, disorders of early postnatal adaptation, and clinical treatment practices. Physicians may have had a lower threshold for initiating empirical antibiotics in preterm or clinically unstable neonates. Respiratory symptoms and systemic instability in these infants may resemble infection and frequently lead to treatment despite the absence of microbiological confirmation [6,7,22].

Reporting treatment frequency alongside blood culture sampling and growth may support antimicrobial stewardship but does not determine which treatment decisions were necessary or which neonates had culture-confirmed EOS.

An important methodological consideration in the interpretation of this retrospective dataset was the distinction between empirical antibiotic treatment, microbial growth in blood cultures, isolates compatible with contamination, and microorganisms recovered from non-sterile sites. These categories were analyzed separately because they do not represent equivalent evidence of invasive infection. In particular, the absence of Gram-negative organisms in blood cultures should not be interpreted as evidence against their established role in EOS, because the number of positive cultures was too small for pathogen-distribution analysis.

The small number of positive blood cultures in this cohort may reflect several factors. Maternal GBS screening and IAP have substantially reduced the incidence of early-onset GBS disease at the population level [1,3,17,18,19]. In addition, the diagnostic yield of neonatal blood cultures may be affected by blood sample volume, timing of sampling, and maternal intrapartum antibiotic exposure [6,20,21]. However, the present study did not include a pre-IAP comparator and was not designed to estimate the effectiveness of prophylaxis. Accordingly, the small number of positive cultures cannot be attributed directly to IAP.

Although Gram-negative organisms, including *Escherichia coli* and *Klebsiella spp*., were frequently isolated from non-sterile neonatal sites, particularly rectal swabs, these findings were analyzed separately from blood culture results because they do not represent evidence of invasive infection. Selective sampling from non-sterile sites may increase the detection of colonizing organisms, including ESBL-producing *Escherichia coli* [31].

Differences between universal screening and risk-based strategies, as well as variation in antibiotic choice and timing, may limit the comparability of findings across populations [3,18,19]. In the present cohort, the no-IAP subgroup among GBS-positive mothers was very small, so the analysis of timing-defined IAP completeness was descriptive only and cannot be used to estimate the effectiveness of prophylaxis. These comparisons also cannot establish a dose–response relationship or equivalence to penicillin-based prophylaxis.

The CRP and PCT analyses are hypothesis-generating and ancillary to the main question of empirical antibiotic initiation. Measurements were available in only 132 and 332 neonates, respectively, so the findings reflect selection for testing rather than systematic assessment of the cohort. Restricting comparisons to neonates without treatment or a recorded infection diagnosis does not remove selection bias and may further alter the composition of the groups. PCT has a physiological postnatal rise during the first 24–48 h of life, and the fixed threshold used here did not account for exact postnatal age at sampling [20,21]. Differences in sampling age, gestational age, clinical condition, or indications for testing could therefore contribute to the observed PCT difference; residual confounding cannot be excluded. Neither the nominally significant PCT result nor the non-significant CRP result establishes the presence or absence of a relationship between maternal GBS colonization and neonatal inflammation. Evaluation of such a relationship would require systematic sampling at predefined postnatal ages and analysis accounting for relevant perinatal factors.

Prematurity, respiratory symptoms, low Apgar scores, metabolic abnormalities, and clinical instability requiring NICU admission may reflect non-infectious postnatal adaptation disorders, while pPROM may heighten concern about infection. NICU admission and prolonged hospitalization may also reflect the concurrent or subsequent clinical course, so their temporal and causal relationships with treatment cannot be established here. Broader obstetric-neonatal outcome data support interpreting morbidity within its perinatal context [32]. Similarly, among pregnancies complicated by placenta previa, histopathologically confirmed placenta accreta spectrum was associated with increased odds of delivery-room resuscitation and neonatal oxygen therapy after adjustment for gestational age, illustrating how specific obstetric pathology may influence early respiratory morbidity [33].

This study has several limitations. First, the primary outcome measured empirical antibiotic initiation for suspected EONI, not culture-confirmed EOS. Systematic case-level adjudication was unavailable, and non-infectious causes of respiratory or systemic symptoms, including transient tachypnea of the newborn, respiratory distress syndrome, persistent pulmonary hypertension, and other adaptation abnormalities, could not be uniformly distinguished from infection. Adjustment for selected covariates does not remove residual confounding by illness severity or variation in treatment thresholds; the model estimates associations with prescribing, not causal effects on EOS. The limited number of treatment events and the wide CIs for maternal GBS colonization also limit precision: clinically relevant increases in antibiotic initiation remain compatible with the estimates despite non-significant *p*-values.

Second, microbiological evaluation was limited to 79 of 2010 neonates (3.93%), with nine positive blood cultures. Sampling was clinically selective, and eight treated neonates had no blood culture obtained. Several isolates were compatible with contamination, so the nine positive cultures cannot be interpreted as nine cases of culture-confirmed EOS. These limitations prevent reliable classification of culture-confirmed EOS and make the sampled subgroup unsuitable for estimating EOS incidence or pathogen distribution in the overall cohort. In particular, no GBS isolates were detected among the sampled neonates, but unsampled neonates cannot be assumed to have been free of GBS EOS. The absence of cultures in eight treated neonates and incomplete sampling and adjudication information also prevent assessment of whether negative or positive culture findings could reliably guide discontinuation or continuation of empirical therapy [26,29].

Third, non-sterile-site isolates were analyzed descriptively and should not be interpreted as clinically equivalent to invasive infection. The study was not designed as a neonatal microbiome study and cannot determine whether IAP modified neonatal colonization patterns. Antimicrobial susceptibility data were not systematically available and were not sufficiently complete or standardized to evaluate whether IAP exposure was associated with changes in neonatal antimicrobial resistance profiles. In addition, incomplete individual-level documentation of actual cefuroxime dosing, repeat doses, allergy-related modifications, and protocol adherence limits characterization of maternal antibiotic exposure. Finally, the single-center retrospective design and local cefuroxime-based IAP protocol may limit external generalizability. Effects of intrapartum antibiotics on early-life microbiome development remain an important topic for future prospective research [23].

Prospective multicenter cohort studies with variables defined a priori, standardized blood culture volume and timing, time-to-positivity assessment, repeat cultures, complete case-level adjudication, detailed maternal antibiotic exposure, complete antimicrobial susceptibility testing, and standardized longitudinal microbiome sampling would be required to assess the effect of IAP on culture-confirmed EOS incidence, pathogen distribution, antimicrobial resistance, and neonatal microbiome composition.

## 5. Conclusions

In this single-center retrospective cohort, empirical systemic antibiotics for suspected EONI were initiated within the first 72 h of life in 71 of 2010 neonates (3.53%). This is a treatment frequency, not an estimate of EOS incidence. The study did not detect a statistically significant association between maternal GBS colonization and antibiotic initiation. However, the RR of 1.16 (95% CI 0.67–2.01) and adjusted OR of 1.33 (95% CI 0.68–2.60) remain compatible with clinically relevant increases in treatment. These results do not establish the absence of an association with antibiotic initiation or an association between maternal GBS colonization and EOS.

No GBS isolates were detected among the sampled neonates; this does not establish the absence of GBS EOS in the overall cohort. Selective sampling and incomplete microbiological information preclude reliable estimates of culture-confirmed EOS incidence or pathogen distribution.

These findings may provide a benchmark for empirical antibiotic prescribing practices in neonatal units and inform the design of prospective studies evaluating strategies to optimize antibiotic stewardship in newborn care.

## Figures and Tables

**Table 1:** Maternal, obstetric, and neonatal characteristics according to maternal GBS colonization status.

Variable	GBS-Positive (*n* = 402)	GBS-Negative (*n* = 1608)	*p*-Value
Maternal age, years	32.8 ± 5.0	32.8 ± 4.8	0.824
Maternal body weight, kg	66.3 ± 12.9	66.2 ± 13.3	0.676
Gestational age at delivery, weeks	38.7 ± 1.3	38.7 ± 1.4	0.904
Preterm birth < 37 weeks	19/402 (4.7%)	94/1608 (5.8%)	0.383
Birth weight, g	3383 ± 469	3375 ± 487	0.793
Apgar score at 1 min	10 (9–10)	10 (9–10)	0.121
Apgar score at 10 min	10 (10–10)	10 (10–10)	0.658
Umbilical cord pH < 7.25	17/399 (4.3%)	46/1606 (2.9%)	0.152
Gestational diabetes mellitus	56/402 (13.9%)	236/1608 (14.7%)	0.704
Pregestational diabetes	1/402 (0.2%)	14/1608 (0.9%)	0.330
Any diabetes	57/402 (14.2%)	250/1608 (15.5%)	0.495
Chronic hypertension	5/402 (1.2%)	31/1608 (1.9%)	0.355
Gestational hypertension	23/402 (5.7%)	70/1608 (4.4%)	0.243
Preeclampsia	6/402 (1.5%)	17/1608 (1.1%)	0.436
Any hypertensive disorder	30/402 (7.5%)	101/1608 (6.3%)	0.391
PROM	37/402 (9.2%)	115/1608 (7.2%)	0.164
pPROM	11/402 (2.7%)	42/1608 (2.6%)	0.889
Rupture-to-delivery interval ≥ 18 h	3/401 (0.7%)	6/1606 (0.4%)	0.395
Suspected intrauterine infection	19/402 (4.7%)	65/1608 (4.0%)	0.540
Cesarean delivery	219/401 (54.6%)	907/1608 (56.4%)	0.518
Neonatal respiratory disorders	38/401 (9.5%)	138/1605 (8.6%)	0.578
NICU admission	20/402 (5.0%)	81/1607 (5.0%)	0.957
Length of hospitalization, days	3 (3–4)	3 (3–4)	0.245

**Table 2:** Systemic antibiotic initiation for suspected EONI according to maternal GBS colonization status.

Maternal GBS Status	Total N	Antibiotic Initiation for Suspected Infection n (%)	RR (95% CI)	*p*-Value
GBS-positive	402	16 (3.98%)	1.16 (0.67–2.01)	0.59
GBS-negative	1608	55 (3.42%)	Reference	—
Total	2010	71 (3.53%)	—	—

**Table 3:** Systemic antibiotic initiation for suspected EONI according to timing-defined IAP completeness among GBS-colonized mothers.

IAP Status Among GBS-Positive Mothers	Total N	Antibiotic Initiation for Suspected Infection n (%)
Complete IAP	232	9 (3.9%)
Incomplete IAP	160	5 (3.1%)
No IAP	10	2 (20.0%)
Total	402	16 (4.0%)

EONI, early-onset neonatal infection.

**Table 4:** Maternal and perinatal characteristics, clinical evaluation, and subsequent course according to systemic antibiotic initiation for suspected EONI.

Variable	Empirical Antibiotics Initiated (n = 71)	No Empirical Antibiotic Initiation (n = 1939)	*p*-Value
A. Maternal and obstetric characteristics and neonatal status at birth			
GBS-positive status	16/71 (22.5%)	386/1939 (19.9%)	0.587
Maternal age, years	33.4 ± 4.5	32.7 ± 4.9	0.224
Maternal body weight, kg	70.7 ± 20.6	66.1 ± 12.9	0.068
Gestational age at delivery, weeks	35.6 ± 3.1	38.8 ± 1.1	<0.001
Preterm birth < 37 weeks	41/71 (57.7%)	72/1939 (3.7%)	<0.001
Birth weight, g	2725 ± 810	3401 ± 450	<0.001
Apgar score at 1 min	8 (7–10)	10 (9–10)	<0.001
Apgar score at 10 min	9 (9–10)	10 (10–10)	<0.001
GDM	14/71 (19.7%)	278/1939 (14.3%)	0.206
Pregestational diabetes	0/71 (0.0%)	15/1939 (0.8%)	1.000
Any diabetes	14/71 (19.7%)	293/1939 (15.1%)	0.289
Any hypertensive disorder	10/71 (14.1%)	121/1939 (6.2%)	0.022
PROM	3/71 (4.2%)	149/1939 (7.7%)	0.279
pPROM	15/71 (21.1%)	38/1939 (2.0%)	<0.001
Rupture-to-delivery interval ≥ 18 h	1/71 (1.4%)	8/1936 (0.4%)	0.277
Suspected intrauterine infection	6/71 (8.5%)	78/1939 (4.0%)	0.118
Cesarean delivery	46/71 (64.8%)	1080/1938 (55.7%)	0.131
Umbilical cord pH < 7.25	6/71 (8.5%)	57/1934 (2.9%)	0.022
B. Clinical presentation, diagnostic evaluation, and subsequent course			
Neonatal respiratory disorders	59/71 (83.1%)	117/1935 (6.0%)	<0.001
NICU admission	52/71 (73.2%)	49/1938 (2.5%)	<0.001
Blood culture obtained	63/71 (88.7%)	16/1939 (0.8%)	<0.001
Positive blood culture among those sampled	7/63 (11.1%)	2/16 (12.5%)	1.000
Neonatologist-diagnosed infection	70/71 (98.6%)	15/1938 (0.8%)	<0.001
Length of hospitalization, days	11 (8–23)	3 (3–4)	<0.001

Table 4 is grouped by clinical context. Section A describes maternal and obstetric characteristics and neonatal status at birth; neonatal birth assessments are not necessarily temporally independent of the treatment decision. Section B includes clinical findings and care processes potentially occurring around or after antibiotic initiation. Respiratory disorders, NICU admission, and blood culture sampling may contribute to, accompany, or follow that decision; positive culture results and recorded clinical diagnoses describe diagnostic evaluation, and length of hospitalization reflects subsequent course. Section B comparisons should not be interpreted as conventional baseline risk-factor analyses or as establishing temporal or causal relationships with treatment. EONI, early-onset neonatal infection.

## Data Availability

The dataset supporting this study has been deposited in a public repository and is accessible at the following URL: https://doi.org/10.17605/OSF.IO/AGY5D (accessed on 27 April 2026).
